# Response to Pulmonary Rehabilitation in Older People with Physical Frailty, Sarcopenia and Chronic Lung Disease

**DOI:** 10.3390/geriatrics2010009

**Published:** 2017-01-22

**Authors:** Luke Attwell, Michael Vassallo

**Affiliations:** 1 Royal Bournemouth & Christchurch Hospitals NHS Foundation Trust, Castle Lane East, Bournemouth BH7 7DW, UK; michael.vassallo@rbch.nhs.uk; 2 Bournemouth University, Poole BH12 5BB, UK

**Keywords:** frailty, sarcopenia, chronic obstructive pulmonary disease, pulmonary rehabilitation

## Abstract

Frailty and sarcopenia are two important clinical syndromes associated with the ageing process, with a high risk of morbidity and mortality. Patients with chronic disease have been shown to have an accelerated decline into a frail state, with patients with both chronic lung disease and frailty having a higher mortality than those with frailty alone. Pulmonary rehabilitation has been found to be an effective intervention in patients with chronic obstructive pulmonary disease (COPD), yet the effect of frailty on this as intervention remains unclear. A narrative literature search of PubMed, Medline complete and the Cochrane library was performed by the reviewers using predefined criteria. Only 3 studies met the selection criteria and were reviewed. These studies highlighted that, although completion rates are lower in patients with both COPD and frailty, pulmonary rehabilitation remains effective as an intervention in this subgroup of patients, with up to 61% of frail patients no longer meeting frailty criteria after completion of a pulmonary rehabilitation programme.

## 1. Introduction

Frailty is a clinical syndrome in which there is a multisystem decline leading to reduced physiological reserve and susceptibility to morbidity and mortality following minor stressor events [1], associated with ageing. It is said to affect 1 in 10 of those over 65 years of age [2] and is associated with disability, hospitalization and death [3]. Frailty is characterized by wasting (reduced strength, muscle mass and weight loss), loss of endurance, loss of balance and mobility, slowing, relative inactivity and reduced cognitive function. The pathophysiology is not fully understood, but is felt to be related to systemic inflammation, malnutrition, endocrine dysfunction, cardiovascular and cerebrovascular disease and neuropsychological impairment [4].

Early identification of frailty is important, as this allows for instigation of interventions that aim to minimize physical decline and reduce hospital admission and morbidity/mortality [5]. These interventions are often multi-faceted and include physical, nutritional, cognitive and educational components.

Patients with chronic disease have been shown to have an accelerated rate of decline into a frail state yet the relationship between chronic lung disease and frailty is not yet fully understood [5], although self-reported frailty makers are higher in those with chronic obstructive pulmonary disease (COPD) than without [6]. People with COPD have deficits in muscle strength and impaired functional status, increasing the risk of them developing frailty. Patients with COPD who are frail also have a higher mortality than those without frailty [7] and are more likely to have sarcopenia and increased morbidity [8], and more severe disease. One study in a UK inner-city hospital found a prevalence of sarcopenia of 14.5% in those with COPD [8]. Frailty and respiratory disease share both risk factors (smoking, ageing) and causative mechanisms (endocrine dysfunction and inflammatory cytokines), and have been shown to be strongly associated with one another [9].

Pulmonary rehabilitation has been shown to markedly improve the symptoms and quality of life of patients with respiratory disease. It relieves dyspnoea and fatigue, as well as increasing exercise tolerance, emotional function and patient’s feeling of control over their illness [10]. As well as improving respiratory symptoms and function, pulmonary rehabilitation programmes also target elements of frailty such as weakness, inactivity and fatigue [8,11]. Pulmonary rehabilitation would therefore be expected to improve frailty, but there is little published data on this as an intervention.

This review aims to assess the interplay between chronic lung disease, frailty and sarcopenia and to address whether pulmonary rehabilitation has any prognostic or symptomatic benefit for patients with chronic lung disease and a frailty syndrome.

## 2. Methods

The initial search strategy was as follows: (frailty or sarcopenia or sarcopaenia) and (chronic obstructive pulmonary disease or copd or lung disease) and (pulmonary rehabilitation). The following databases were searched: PubMed, the Cochrane library and Medline complete. This initial search produced 145 full text articles. The researcher also used additional texts from reference lists and Google scholar to broaden the search. Keywords used throughout the literature search included chronic obstructive pulmonary disease, frailty, sarcopenia and pulmonary rehabilitation. MeSH terms were not used.

The criteria used for including studies in this review are as follows:

### 2.1. Types of Studies

Published research that investigated the relationship between patients with chronic lung disease and frailty or sarcopenia and outcomes of pulmonary rehabilitation compared with patients without a frailty syndrome. Ideally randomized controlled trials (RCTs) would be included, but no such articles were found.

### 2.2. Types of Participants

Older people with a chronic lung disease with or without frailty undergoing a pulmonary rehabilitation programme.

### 2.3. Types of Intervention

Completion of a pulmonary rehabilitation programme of varying length and intensity. Duration varied from 4 to 18 weeks, a session’s length of between one to two hours, with up to two sessions a week.

### 2.4. Types of Outcome

Primary: Reduction in markers of frailty or sarcopenia in patients completing pulmonary rehabilitation.

Secondary: Improvements in quality of life, functional status and performance.

A total of three studies were reviewed. Themes that were reviewed include the relationship between sarcopenia/frailty and outcomes following pulmonary rehabilitation, completion rates of pulmonary rehabilitation and the patient demographics of those undertaking pulmonary rehabilitation.

## 3. Studies

No randomized control trials were found. Combinations of observational and case control studies were included.

Maddocks et al. (2016) undertook a prospective cohort study over a four-year period looking into pulmonary rehabilitation in frail patients with COPD [5]. The pulmonary rehabilitation programme consisted of an 8-week outpatient exercise and education programme with 2 supervised and 1 home based session. Supervised sessions comprised 1 h of exercise and 45 min of education.

25.6% of 816 participants of the pulmonary rehabilitation programme were deemed frail (according to the Fried phenotype model), whilst only 10% did not meet any of the frailty criteria. Of all the criterion, exhaustion was met the most (65.3%) followed by weakness, reduced activity and slowness. Frailty was more common amongst women than men; with an increased prevalence as the Medical Research Council (MRC) dyspnoea scale increases (a three-fold increase from a MRC score of 3 to 5).

Frail patients were, however, less likely to complete the pulmonary rehabilitation programme (55% compared with 75.5% of pre-frail and 84.1% of non-frail; see Table 1). This was mainly due to either deterioration in their frail state or admission to hospital. Statistically significant improvements were reported amongst many domains including MRC dyspnoea scale, handgrip, Chronic Respiratory Questionnaire (CRQ) fatigue, emotional and mastery score, Hospital Anxiety and Depression Score (HADS) and the incremental shuttle walk test (ISWT). Of the one hundred and fifteen frail patients who completed the pulmonary rehabilitation programme, seventy one (61.7%) were pre-frail or robust following completion.

Another paper by Jones et al. (2015) researched the interaction between sarcopenia, COPD and the response to pulmonary rehabilitation [8]. The study recruited six hundred and twenty-two patients with stable COPD. The European Working Group on Sarcopenia in Older People (EWGSOP) guidelines were used & found a prevalence of sarcopenia at 14.5%. This increased with age and GOLD (Global Initiative on Obstructive Lung Disease) stage, with a two-fold increase from stage 2 to 4. For a diagnosis of sarcopenia to be made, both low muscle mass and muscle function [12]. The pulmonary rehabilitation programme also consisted of an 8-week outpatient exercise and education programme with 2 supervised and 1 home based session with supervised sessions comprising 1 h of exercise and 45 min of education.

Completion of pulmonary rehabilitation was the same amongst those with and without sarcopenia (81% in both groups). 28% of patients with sarcopenia following rehabilitation no longer met EWGSOP criteria. They did report no difference in sarcopenia prevalence between patients with and without quadriceps weakness; highlighting that sarcopenia may be an entity that cannot be diagnosed solely based on generalized wasting or weakness. A large number of participants (315 of 554) had quadriceps weakness, yet only 47 of those with quadriceps weakness also met the EWGSOP criteria for a diagnosis of sarcopenia, whilst a further 33 met EWGSOP criteria without quadriceps weakness. Pulmonary rehabilitation was found to reverse sarcopenia in patients with a low skeletal muscle index (SMI) or functional performance that was close to the cut-off. Patients with COPD and sarcopenia responded well to pulmonary rehabilitation, with improvements reported in exercise capacity, functional performance, limb strength and health status, similar to patients without sarcopenia.

A third, small study by Mittal et al. looked at the effect of pulmonary rehabilitation on gait speed and frailty status (using Fried’s criteria) [13]. The pulmonary rehabilitation programme is stated to have lasted between 60 and 90 min, but the study does not clarify the duration of the programme. The study population included 41 patients with a chronic lung disease, including COPD, asthma, interstitial lung disease and pulmonary hypertension. There was a high non-completion rate, with only 30 (73.2%) of participants competing the 12-week course. At commencement of the study, 17% of patients were frail, 61% pre-frail and 22% robust.

The study reported fewer frail patients after the six weeks of rehabilitation, with only 7% meeting the frailty criteria. This was not, however, statistically significant. 5 patients had deterioration in their frailty status. Gait speeds increased over the 6-week programme, from 52.9 to 61.1 m (*p* = 0.001) [13].

### 3.1. Frailty & Sarcopenia Definition

There have been many definitions and scoring systems of frailty, with a growing consensus that components include reductions in body mass, strength, endurance, balance, exercise tolerance and reduced activity [4]. An important study looking into common frailty characteristics by Fried et al. defined frailty as an entity independent from medical co-morbidity and disability. They stated that a diagnosis of a frailty syndrome requires 3 of the following criteria [4]:

(1) “Shrinking: unintentional weight loss of ≥10 pounds in the prior year or, at follow-up, of ≥5% of body weight in prior year (by direct measurement of weight).
(2) Weakness: grip strength in the lowest 20% at baseline, adjusted for gender and body mass index.
(3) Poor endurance and energy: as indicated by self-report of exhaustion. Self-reported exhaustion, identified by two questions from the CES–D scale [14], is associated with stage of exercise reached in graded exercise testing, as an indicator of VO_2_ max, and is predictive of cardiovascular disease.
(4) Slowness: The slowest 20% of the population was defined at baseline, based on time to walk 15 feet, adjusting for gender and standing height.
(5) Low physical activity level: A weighted score of kilocalories expended per week was calculated at baseline [15], based on each participant’s report. The lowest quintile of physical activity was identified for each gender” [4].

Markers of frailty may be an interesting way to classify patients with chronic lung disease, as it is associated with deficits that alter prognosis—such as muscle strength, inactivity and reduced physiological reserve [16]. It is accepted that physical exercise training involving strength training, balance and coordination & movement speed has been shown to improve frailty markers in multiple randomized control trials [17], however, there has been little research into the effect of a pulmonary rehabilitation programme on frailty markers in patients with underlying lung disease.

Skeletal muscle dysfunction, in addition to smoking, is a key contributor to the development of COPD, and is the basis for pulmonary rehabilitation, to improve skeletal muscle function and exercise tolerance.

### 3.2. Frailty and Respiratory Impairment

Frailty and respiratory impairment have been shown to be strongly associated with one another. As patients with COPD deteriorate, they become increasingly inactive, lose muscle strength and subsequently develop sarcopenia and frailty. Those with lung disease also experience problems with mobility, reduced gait speed and falls, contributing to the high prevalence of frailty in COPD [18]. Shortness of breath has also found to be a strong predictor of frailty [19]. Up to 94% of patients with COPD experience dyspnoea, which significantly affects their quality of life and activities of daily living, and leads to inactivity. One study found that the prevalence of frailty was higher amongst those with COPD than the general population [9].

Work by Vaz Fragoso using data collected in the Cardiovascular Health Study investigated the relationship between frailty and respiratory impairment [9]. They found that patients who met criteria for pre-frailty or frailty had a statistically significant 62% and 88% increase in the odds of having obstructive airflow limitation and 80% and 205% increase in having a restrictive pattern disorder [9]. Over the four years of the study, those with frailty features had a statistically significant 42% increased odds of developing respiratory impairment (based on age adjusted spirometry results). Those with respiratory impairment had a statistically significant increase in odds of developing frailty by 58%. Those patients who were frail with respiratory impairment had the highest mortality; at a 2.5 fold increase compared to those who were not frail without respiratory impairment. The effects of frailty and respiratory impairment were found to be multiplicative, not additive, with *p* = 0.037 [9].

The mechanisms responsible for the interplay between frailty and COPD are not yet fully understood, but may well involve impaired innate immune responses [20]. Both frailty syndromes and COPD are associated with increased systemic inflammation, highlighted by increased serum levels of inflammatory markers including the white blood cell count, C-reactive protein, tumour necrosis factor-alpha and interleukin 6. Raised titers of interleukin 6 in particular have been associated with anaemia, reduced muscle mass and sarcopenia, and has an inverse correlation with circulating anabolic hormones such as insulin like growthfactor-1 and dehydroepiandrosterone sulfate in patients with frailty [21]. Patients with COPD are likely to also have a persistent state of chronic inflammation and thus particularly prone to developing frailty.

It would appear therefore, that patients who are frail are at increased risk of developing respiratory impairment, and patients with underlying lung disease are more likely to be or become frail. This leads to thoughts on how best to manage this high-risk group of patients with a high morbidity and mortality.

### 3.3. Pulmonary Rehabilitation

The American College of Chest Physicians first defined pulmonary rehabilitation in 1974. It is a structured programme of exercise and education used in patients with chronic respiratory disease, most commonly COPD, to minimize symptoms and improve quality of life [22]. It consists of two sessions a week for six to eight weeks with 30 min of physical activity per week in parallel. Trained professionals in a group setting (the British Thoracic society recommends a ratio of 1:8) deliver a combination of continuous and interval aerobic training and progressive muscle resistance training to both upper and lower limb extremities. It has been clinically proven to be an effective intervention in improving symptoms and quality of life of patients with COPD.

The aerobic training aims to increase inspiratory volume and reduce hyperinflation, subsequently improving dyspnoea on exertion. It also increases muscle function, increasing exercise tolerance and delaying fatigue. The education targets self-management of the patients’ condition and elicit behavioural change through problem solving, decision making and goal setting [23], to alter smoking habits, nutrition, medication compliance and breathing techniques.

## 4. Conclusions and Outlook

Pulmonary rehabilitation has been shown to be an effective intervention in improving symptoms, quality of life and functionality in patients with COPD. Studies have found a strong association between both the aetiology and prevalence of frailty, sarcopenia and chronic respiratory disease, with a large proportion (up to a quarter) of patients with COPD also meeting the criteria for frailty. This cohort of patients is at a higher risk of morbidity and mortality and is difficult to manage. What has been consistently demonstrated is that these patients are less likely to participate and complete a pulmonary rehabilitation programme (see Table 1); however, when they do outcomes are favorable. Up to 61% of frail participants no longer met Fried’s criteria following pulmonary rehabilitation completion. Improvements were reported across domains, including gait speed, grip strength, dyspnoea as well as improvements in self-reported quality of life, anxiety and depression scores.

A limitation of the review is the difference in length, duration and intensity of pulmonary rehabilitation programmes. Although the studies seem to agree that a pulmonary rehabilitation programme improves markers of frailty and sarcopenia in patients with chronic lung disease, it remains unclear whether the characteristics of the programme affect outcomes. This could be looked at in more detail.

Although it would seem clear that there is an association between frailty, sarcopenia and chronic lung disease, and that mortality is increased, the cause of this remains unclear. There is, however, limited research into pulmonary rehabilitation as an intervention for frailty. Further work in this area could focus on the reasons behind poor rates of completion of pulmonary rehabilitation amongst the frail & sarcopenic population, and how to increase this. Studies could also look at longer term benefits of the intervention and whether patients later fall back into the frail category. There might also be a role for frailty specific pulmonary rehabilitation programmes in the future.

## Figures and Tables

**Table 1:** Completion rates of pulmonary rehabilitation programmes.

Study	Completion Rates		
	Total	Frail	Non-Frail
Maddocks et al. [5]	70.3%	55%	79.80%
Jones et al. [8]	81.0%	81%	81%
Mittal et al. [13]	73.2%	did not distinguish between frail and non-frail	
